# Melanoma Genetics/Genomics

**DOI:** 10.3389/fonc.2013.00309

**Published:** 2013-12-17

**Authors:** Michael R. Eccles

**Affiliations:** ^1^Developmental Genetics and Pathology Laboratory, Department of Pathology, Dunedin School of Medicine, University of Otago, Dunedin, New Zealand

**Keywords:** melanoma, therapeutic targets, biomarkers, genetic pathways, personalized medicine

Gene mutations represent a major driving force in the onset and progression of melanoma. Consequently many genes are being investigated for their role in melanomagenesis, including not only inherited genes but also genetic defects that are acquired due to environmental factors, such as excessive sun exposure. The field of melanoma genetics thus encompasses genes in familial melanoma through to non-inherited genes that increase risk of melanoma. Melanoma genomics on the other hand is the study of genomes of melanoma cells and other cell types and their role in melanoma onset and progression. A “genome” includes not only all the genes of a cell, but also any genetic factors involved in programing the cell and its function.

The present volume aims to provide the reader with a snapshot of current genetic and genomic investigations of melanoma, with special emphasis on targeted treatments, and personalized medicine. A collection of Opinion, Review, Primary Research, Hypothesis and Theory, and Methods articles has been assembled that describes the panoply of genes, therapeutic targets, biomarkers, genetic pathways, and pathogenic mechanisms involved in melanoma onset and metastasis, and of clinical outcomes in patient in response to chemotherapy, immunotherapy, and personalized treatment options.

Much progress has been made in identifying individual genes and pathways involved in melanomagenesis, as outlined in the Review Article by Wangari-Talbot and Chen (1). Indeed, the discovery that melanomas frequently contain somatically acquired mutations in the *BRAF* gene that drive melanoma growth has revolutionized melanoma treatment options, and led to the development of personalized targeted treatments for patients with metastatic melanomas bearing a *BRAF* mutation, reviewed by Klinac et al. (2).

Despite melanomas harboring somatically acquired mutations in genes like *BRAF* or *NRAS*, the response of individual melanoma patients to BRAF inhibitor treatments is very variable, and Stones et al. (3) have investigated gene mutation status with respect to sensitivity to BRAF inhibitors and combination targeted therapies in a panel of New Zealand human melanoma cell lines in their Original Research Article.

Mutations in genes like *GNAQ*, *GNA11*, and *BAP1* are associated with uveal melanoma or blue nevi, and for the first time Hawkes et al. (4) have investigated in their Original Research Article whether inherited mutations in these genes are associated with familial predisposition to uveal melanoma or blue nevi.

Although *BRAF* mutations can be identified from the very earliest stages of melanoma onset, targeted BRAF inhibitor therapies are presently validated for use in advanced stage IV melanomas. Could therapies targeting *BRAF* be successfully used to treat earlier stages of melanoma? This is the subject of an Opinion Article by Ahn and Eccles (5).

With the plethora of genomic information, treatments, and outcome data available from melanoma studies, what is the best way to manage and interrogate all of this burgeoning information? Trevarton et al. (6) describe a web tool integrating multiple sources of genomic information called MelanomaDB in their Methods Article. Then immediately following this is a critique by Reinhold (7) of the advantages and disadvantages of the approach taken by the MelanomaDB article for data integration.

In addition to “driver” mutations in *BRAF*, and the related growth promoting pathways, other pathways are also very likely to be important in melanoma metastasis, including the Hippo pathway, which is discussed in the Hypothesis and Theory Article by Kim et al. (8).

An Original Research Article by Kim et al. (9) investigates the role of epithelial-mesenchymal transition marker expression in human melanocytes and melanoma cell lines. In a similarly themed article Eccles et al. (10) suggest that switching of melanoma cells from a proliferative to an invasive phenotype during metastasis has parallels with developmental mechanisms, which could be under genetic control. They propose a genetic switch theory, which they hypothesize is involved in the transition of melanoma cells to an invasive phenotype in their Hypothesis and Theory Article.

Biomarkers of melanoma progression and metastasis are expected to help with further stratification of patients with poor prognosis following melanoma diagnosis, as discussed by Dye et al. (11) in their Review Article. Expression of one factor called GLIPR1 was found to correlate with the invasive potential in melanoma cells, as demonstrated in an Original Research Article by Awasthi et al (12).

Metastasis generally involves the dissemination of circulating melanoma cells, as discussed in an Opinion Article by Joshi et al. (13), but frequently melanomas metastasize to the brain, which is discussed in an Opinion Article by Yashin et al. (14). The potential for targeted therapy of melanoma brain metastasis through *in vivo* modeling and molecular characterization is the subject of a Review Article by Gaziel-Sovran et al. (15).

This collection of articles clearly demonstrates the impact that melanoma genetics and genomics has had on targeted treatments and improved outcomes of melanoma patients in the past decade, and of the promise yet to come, but melanoma remains an important public health issue in Western societies. This is especially so in New Zealand and Australia, where the recorded incidence rates are the highest in the world (41.2 per 100,000 population in New Zealand, age standardized to the Segi world population, 2004, and 37.2 per 100,000 in Australia, as compared to, for example, 11.9 per 100,000 in Western Europe. Clearly much work still needs to be done to address these high incidence and mortality rates of melanoma.
